# Differences in metabolic profiles between the Burmese, the Maine coon and the Birman cat—Three breeds with varying risk for diabetes mellitus

**DOI:** 10.1371/journal.pone.0249322

**Published:** 2021-04-22

**Authors:** Malin Öhlund, Elisabeth Müllner, Ali Moazzami, Ulrika Hermansson, Ann Pettersson, Fredrick Anderson, Jens Häggström, Helene Hansson-Hamlin, Bodil S. Holst

**Affiliations:** 1 Department of Clinical Sciences, Swedish University of Agricultural Sciences, Uppsala, Sweden; 2 Department of Molecular Sciences, Swedish University of Agricultural Sciences, Uppsala, Sweden; 3 University Animal Hospital, Swedish University of Agricultural Sciences, Uppsala, Sweden; 4 Department of Medical Biosciences, Umeå University, Umeå, Sweden; University of Illinois, UNITED STATES

## Abstract

Feline diabetes mellitus shares many features with type 2 diabetes in people, regarding clinical presentation, physiology, and pathology. A breed predisposition for type 2 diabetes has been identified, with the Burmese breed at a fivefold increased risk of developing the condition compared to other purebred cats. We aimed to characterize the serum metabolome in cats (n = 63) using nuclear magnetic resonance metabolomics, and to compare the metabolite pattern of Burmese cats with that of two cat breeds of medium or low risk of diabetes, the Maine coon (MCO) and Birman cat, respectively. Serum concentrations of adiponectin, insulin and insulin-like growth factor-1 were also measured (n = 94). Burmese cats had higher insulin and lower adiponectin concentrations than MCO cats. Twenty one metabolites were discriminative between breeds using a multivariate statistical approach and 15 remained significant after adjustment for body weight and body condition score. Burmese cats had higher plasma levels of 2-hydroxybutyrate relative to MCO and Birman cats and increased concentrations of 2-oxoisocaproic acid, and tyrosine, and lower concentrations of dimethylglycine relative to MCO cats. The metabolic profile of MCO cats was characterized by high concentrations of arginine, asparagine, methionine, succinic acid and low levels of acetylcarnitine while Birman cats had the highest creatinine and the lowest taurine plasma levels, compared with MCO and Burmese. The pattern of metabolites in Burmese cats is similar to that in people with insulin resistance. In conclusion, the metabolic profile differed between healthy cats of three breeds. Detection of an abnormal metabolome might identify cats at risk of developing diabetes.

## Introduction

Diabetes mellitus (DM) is an increasingly common endocrinopathy in cats and has many features in common with human type 2 diabetes (T2DM) [1, 2]. Cats and humans share many risk factors for developing the disease, for example, the association with insulin resistance (IR) coupled to obesity and a sedentary lifestyle, and β-cell loss with amyloid deposition in the islets of Langerhans in the pancreas [3–6]. There is a genetic predisposition in both people [7–9] and cats, with the Burmese cat breed at increased risk [10–13]. An inherited dyslipidemia has been suggested to increase IR and predispose Burmese cats to the disease [14]. Abnormally increased serum triglyceride (TG) concentrations after oral fat tolerance test have been described [15], indicating a delayed clearance of TG compared to unaffected Burmese cats. In people, hypertriglyceridemia has been associated with both IR and T2DM [16–20]. Hypertriglyceridemia is also one of the components of the metabolic syndrome in people, together with central obesity, increased blood pressure and IR [21]. People with metabolic syndrome have a fivefold increased risk of developing T2DM [22]. Subsequent studies have described aberrations in the cholesterol lipoprotein fraction profiles in lean Burmese cats, similar to obese cats, with increases in very low-density lipoprotein (VLDL) concentrations, and decreases in high-density lipoproteins (HDL), a pattern similar to the metabolic syndrome in people [23–26]. In addition, the expression patterns of several genes involved in lipid metabolism as well as low circulating adiponectin concentrations in lean Burmese cats resemble those of obese cats. In people, low adiponectin levels are associated with IR and T2DM [27–31].

Metabolites are small molecules that are chemically transformed in the metabolism and provide functional readouts of cellular biochemical activities. The metabolome provides a measurement of the metabolic phenotype that is a net result of genomic, transcriptomic, and proteomic variability [32]. Metabolomics is useful for studying metabolic diseases, or traits, such as T2DM and IR, especially considering that the more than 100 so far identified T2DM associated gene loci in people have only small to moderate effects on the individual’s susceptibility to the disease [33]. Metabolomics has been utilized in diabetes research in people [34–40], and an abnormal pattern in the branched-chain and aromatic amino acids has been described [41–44]. Few studies using the metabolomics approach are available in cats. The metabolome of cats in remission has been studied and compared to that of control cats, showing an abnormal metabolism in cats in remission [45]. In a PhD thesis the metabolome has been shown to differ between obese and normal weight cats, and between senior cats of Burmese compared to those of other breeds [46]. In studies not related to DM, one study analyzed the urine metabolome using gas chromatography/time-of-flight mass spectrometry in eight healthy domestic cats [47], and in another study the effects of dietary macronutrient composition on the plasma metabolome of healthy adult cats were assessed with liquid chromatography followed by mass spectrometry (LC/MS) [48].

The objective of the present study was to characterize the feline metabolic profile in healthy Burmese cats, and compare it to two cat breeds of medium or low risk for developing DM, the Maine coon (MCO) and Birman cat, respectively, [11] by using nuclear magnetic resonance (NMR) metabolomics, serum biochemistry, and hormone immunoassays. Differences in the metabolome of these three feline breeds might shed light into preventative measures in the future under a clinical setting. The information may be used when metabolome perturbations of a patient begin to emerge and can be compared to known disease associated metabolome profiles.

## Materials and methods

### Study population

The study was approved by the Uppsala Ethical Committee on Animal Research (C 299/12 and C 12/15) and the Swedish Board of Agriculture (31-11654/12), and written informed consent to participate in the study was obtained from all owners.

Healthy, adult (> 1 year), purebred, client-owned cats (n = 106) of three different breeds (Burmese, MCO and Birman) were included in the study. Food was withheld for at least 12 hours prior to sampling. Cats were weighed and a physical examination including body condition scoring using a 9-grade scale [44] was performed by a veterinarian at the University Animal Hospital, Swedish University of Agricultural Sciences, Uppsala, Sweden, or the Bagarmossen Anicura Referral Hospital, Stockholm, Sweden. Of the Burmese cats, 31 cats were sampled in 2013. All other cats were sampled 2015–2016. Cats with a body condition score (BCS) between 3 and 4 were grouped as underweight, 5 were considered normal weight, and cats with a BCS between 6 and 9 were grouped as overweight [47]. Owners completed a questionnaire which included questions concerning the cat’s age, breed, sex, neutering status, any current or previous medications or medical issues, and time of last meal.

Cats were excluded if they were non-fasted, non-compliant at sampling, had a history of or ongoing severe organ related or systemic disease, or if they had received progestin or corticosteroid treatments during the last year. Cats were also excluded if serum biochemistry showed values clearly outside the reference range, although small deviations in fasting serum creatinine levels were accepted [48]. Fasting serum concentrations of creatinine ≤200 μmol/L, alanine aminotransferase (ALAT) ≤2.8 μkat/L, and fructosamine ≤350 μmol/L were accepted.

### Sampling

Blood was drawn from the cephalic vein and collected into serum tubes. Samples were centrifuged for 10 minutes at 3000 rpm and serum was thereafter aliquoted and stored cool and analyzed within 24 hours, or stored in microtubes at -70°C until further analysis.

### Analyses

#### Serum biochemistry

All serum samples were analyzed for ALAT, creatinine, and fructosamine concentrations on an automated chemistry analyzer (Abbott Architect c4000, Abbott Park, IL, USA) at the Clinical Pathology Laboratory, University Animal Hospital, Swedish University of Agricultural Sciences, Uppsala, Sweden.

Lipoprotein profiles were obtained at the department of Medical Biosciences, Umeå University by utilizing an automated HPLC system (Elite LaChrom, Hitachi, Krefeld, Germany) with a Superose 6 size-exclusion column (GE Healthcare, Uppsala, Sweden). Plasma samples were diluted 1:16 in elution buffer that consisted of 10 mM Tris, 150 mM NaCl and 0,02% NaN_3_, and injected into the column. On-line measurements of triglyceride and cholesterol concentrations were performed using appropriate reagents (Roche, Basel, Switzerland). The reagents were diluted 1:2 with lab grade water prior to analyses. As a standard for lipoprotein profiles, a human plasma sample with a known lipid concentration was used. All data was processed using the EZChrom Elite software (Agilent Technologies, Boeblingen, Germany).

Free fatty acids were measured with the MaxDiscovery^™^ Non-esterified fatty acids (NEFA) Assay Kit (Bioo Scientific, Austin TX, US), at the Clinical Sciences laboratory, Swedish University of Agricultural Sciences, Uppsala, Sweden.

#### Hormone immunoassays

Total adiponectin concentrations were assayed using the Adiponectin Human ELISA, High Sensitivity (BioVendor—Laboratorni medicina, Brno, Czech Republic), insulin concentrations were measured with the Mercodia Feline Insulin ELISA (Mercodia AB, Uppsala, Sweden), and insulin-like growth factor (IGF)-1 with the human E20 Insulin-like Growth Factor-I ELISA (Mediagnost, Reutlingen, Germany). All analyses were performed in duplicate at the Department of Clinical Sciences laboratory, Swedish University of Agricultural Sciences, Uppsala, Sweden. All hormonal assays have previously been validated for use in cats [49–52]. If the intra-assay coefficient of variation (CV) was above 10%, samples were rerun, and the highest accepted CV was 11% (one sample). For IGF-1, samples at concentrations above 28 ng/mL on the standard curve were diluted further and rerun, to avoid interference by IGF-binding proteins, which may not be efficiently removed when using the standard protocol [51].

#### NMR-based metabolomics analyses

Metabolomics analyses were performed on a subset of samples, all collected 2015–2016 (in total n = 63; Burmese n = 15, MCO n = 25, Birman n = 23,). Nanosep centrifugal filters with 3-kDa cutoff (Pall Life Science, Port Washington, NY) were washed to remove glycerol from the filter membrane. 60 μl serum were filtered at 10,000 g, 4°C. 40 μl of filtrate were mixed with 50 μl phosphate buffer (0.4 mol/L, pH 7.0), 15 μl D_2_O, 55 μl millipore water, and 10 μl sodium-3-(trimethlsilyl)-2,2,3,3,-tetradeuteriopropionate (TSP, 5.8 mmol/L) (Cambridge Isotope Laboratories, Andover, MA) as an internal standard to be able to quantify metabolites. Analyses were performed on a Bruker spectrometer operating at 600 MHz equipped with a cryogenically cooled probe and auto sampler at the Department of Molecular Sciences, Swedish University of Agricultural Sciences, Uppsala, Sweden. The ^1^H NMR spectra were obtained using zgesgp pulse sequence (Bruker Spectrospin Ltd) at 25°C with 512 scans at 65,356 data points over a spectral width of 17,942.58 Hz (acquisition time: 1.83 s, relaxation delay 4 s). Baseline and spectral phase correction were performed manually using Chenomx. The line width was adjusted to 1.1 Hz for all spectra. Fifty-eight metabolites were identified and their concentrations were calculated using an automated quantification algorithm (AQuA) accounting for interfering signals as previously described [53].

### Statistical analysis

Normally distributed data are reported as mean with standard deviation (SD), and non-normally distributed data as median with interquartile range (IQR). A one-way ANOVA was used to compare age, sex, body weight (BW) and BCS between breeds. The assumption of normally distributed residuals and equal variances in the model was examined by visual inspection of diagnostic plots (histogram of residuals and normal probability plots of residuals). If residuals were not normally distributed, data were log-transformed and diagnostic plots were reevaluated. If residuals remained non-normally distributed, a non-parametric test was used.

To assess differences in metabolites between breeds, univariate statistical analyses were performed (Minitab, version 17.3.1) on metabolites identified as discriminative via the multivariate approach described below by using one-way ANOVA (for normally distributed data), or Kruskal-Wallis test (for not normally distributed data). Further, the Tukey’s post hoc test was applied to assess differences between the three breeds. To adjust for influence of BW and BCS, a univariate mixed linear regression model was applied, for each metabolite identified as discriminative between breeds (SAS, version 9.4). Concentrations are reported as least square means or geometric means with 95% CI as described above. The significance level was set at *P* < 0.05.

Multivariate regression was used to investigate the effects of breed, BW, and BCS on the concentrations of creatinine, ALAT, fructosamine, VLDL-TG, HDL-cholesterol, FFA, insulin, adiponectin, and IGF-1 using SAS (version 9.4). Potential interactions were controlled for by including interaction factors between the explanatory variables. Concentrations were reported as least square means with 95% CI, and if data were logged for analysis, least square means were back-transformed and reported as geometric means with 95% CI. *P*-values given for the multivariate analyses are based on Wilk’s lambda. Effects of differences in storage time and sample handling were evaluated by *t-test* within the Burmese breed group, where samples were collected during two different time periods (2013, and 2015 to 2016, respectively).

Multivariate statistical analyses were performed on metabolomics data using SIMCA 14 software (Umetrics, Umeå, Sweden). Principal component analysis (PCA) was applied to get an overview of the data and to exclude potential outliers by using the PCA-Hotelling’s T2 Ellipse (95% confidence intervals (CI)). To assess differences between the breeds, partial least square discriminant analysis (PLS-DA) was applied, which can take class membership (e.g. cat breed) into account. To determine discriminative metabolites between the breeds, variable influences on projection (VIP) values were used. Metabolites with VIP values > 1 for which the corresponding jackknife-based 95% CIs were not close to or included zero were considered discriminative. Cross-validated ANOVA was used to confirm validity and reliability of the PLS-DA model. Additionally, R2 (proportion of variation modeled in the component) and Q2 parameters (proportion of variation in the data predictable by the PLS-DA model) are reported.

## Results

### Study population

Out of the 106 recruited cats, 12 were excluded from the study for not having met the inclusion criteria, leaving the study population at 94 cats (46 Burmese, including 31 cats sampled in 2013, 25 MCO, and 23 Birman cats). Reasons for exclusion included non-fasting (n = 3), non-compliance (n = 5), concurrent illness (n = 3), or increased serum biochemistry values (n = 1). Descriptive statistics by breed for the variables age, BW, BCS, and sex distribution are shown in Table 1. Age and sex distribution did not differ between breeds, however, BW (MCO > Burmese > Birman) and BCS (Burmese > Birman) did (Table 1).

**Table 1:** Descriptive statistics of the included cats (n = 94) by breed.

Variable^1^		Burmese (n = 46)	Maine coon (n = 25)	Birman (n = 23)	P-value*
**Age** (years)	Median	5.0 ^a^	8.0 ^a^	6.0 ^a^	0.2
	(IQR)	(2–9)	(3–10)	(2–11)	
**BW** (kg)	Median	4.4 ^a^	5.4 ^c^	3.3 ^b^	<0.001
	(IQR)	(3.6–5.1)	(4.6–6.6)	(3.0–4.1)	
**BCS** (scale 1–9)	Median	6.0 ^a^	5.0 ^a,b^	5.0 ^b^	0.014
	(IQR)	(5.0–6.0)	(5.0–5.0)	(5.0–6.0)	
**Sex** (n)	Male	2 (4%) ^a^	2 (8%) ^a^	3 (13%) ^a^	0.44
	Neutered male	22 (48%)	7 (28%)	5 (22%)	
	Female	9 (20%)	7 (28%)	8 (35%)	
	Neutered female	13 (28%)	9 (36%)	7 (30%)	

IQR, interquartile range; BW, body weight; BCS, body condition score.

^1^ Data are shown as median and interquartile range and number of cats and proportions.

^a,b,c^ Numbers within a row with different superscript letters differ from another at *P* < 0.05.

* P-values from one-way ANOVA.

#### Serum biochemistry and hormonal variables

Effects of breed, BW, and BCS on the concentrations of creatinine, fructosamine ALAT, VLDL-TG, HDL-cholesterol, FFA, adiponectin, insulin, and IGF-1, showed that for all variables except ALAT and FFA, a significant model could be obtained. Breed (*P* < 0.0001) and BW (*P* < 0.0001), but not BCS (*P* = 0.14), had strong overall effects in the multivariate model. There were no significant interactions present between any of the explanatory variables. Results from the multivariate model with breed differences are summarized in Table 2.

**Table 2:** Serum biochemistry and hormonal concentrations by breed in 94 cats.

Analyte	Concentration^1^			
	Burmese (n = 46)	Maine coon (n = 25)	Birman (n = 23)	*P*^2^
**Creatinine** (μmol/L)	128 (119–136) ^a^	128 (117–138) ^a^	163 (152–174) ^b^	<0.0001
**Fructosamine** (μmol/L)	251 (242–260) ^a^	238 (228–249) ^a^	246 (234–257) ^a^	0.004
**ALAT** (μkat/L)	1.1 (0.9–1.2) ^a^	0.8 (0.6–1.0) ^a^	1.2 (1.0–1.4) ^a^	0.110
**VLDL-TG** (mmol/L)	0.19 (0.15–0.23) ^a^	0.10 (0.07–0.12) ^b^	0.23 (0.18–0.30) ^a^	<0.0001
**HDL-cholesterol** (mmol/L)	4.8 (4.4–5.3) ^a^	4.2 (3.7–4.7) ^a^	6.3 (5.6–7.2) ^b^	<0.0001
**FFA** (mmol/L)	0.52 (0.44–0.61) ^a^	0.44 (0.36–0.53) ^a^	0.40 (0.32–0.49) ^a^	0.170
**Adiponectin** (ng/mL)	429 (390–468) ^a^	609 (562–656) ^b^	466 (416–516) ^a^	<0.0001
**Insulin** (ng/mL)	211 (157–283) ^a^	115 (81–164) ^b^	169 (116–247) ^a,b^	0.036
**IGF-1** (ng/mL)	660 (532–818) ^a^	378 (292–489) ^b^	773 (587–1018) ^a^	0.001

ALAT, alanine aminotransferase; VLDL, very low-density lipoprotein; TG, triglycerides; HDL, high-density lipoprotein; FFA, free fatty acids; IGF, insulin-like growth factor.

^1^ Concentrations are shown as estimated least square means for each breed, with 95% confidence intervals (CI). If residuals were non-normally distributed, data were logged for analysis, and least square means were back-transformed and reported as geometric means, with 95% CI.

^2^ *P*-values represent breed differences based on results from the multivariate linear regression model including adjustments for body weight and body condition score.

^a,b^ Numbers within a row with different superscript letters differ from another at *P* < 0.05.

Body weight had significant effects on fructosamine, VLDL-TG, insulin, IGF-1, and adiponectin concentrations. Increasing BW increased concentrations of fructosamine with 8.1 μmol/L per kg (*P* = 0.0015), VLDL-TG with 27% per kg (*P* < 0.0001), insulin with 20% per kg (*P* = 0.024), IGF-1 with 33% per kg (*P* < 0.0001), and decreased the concentration of adiponectin with 40.3 ng/mL per kg (*P* = 0.0003). Overweight cats had 20.3 μmol/L higher average creatinine concentrations than normal weight cats (P = 0.0017).

None of the above mentioned parameters differed between Burmese cat samples collected in 2013 and those collected in 2015 & 2016.

#### Metabolomics data

**Fig 1:**
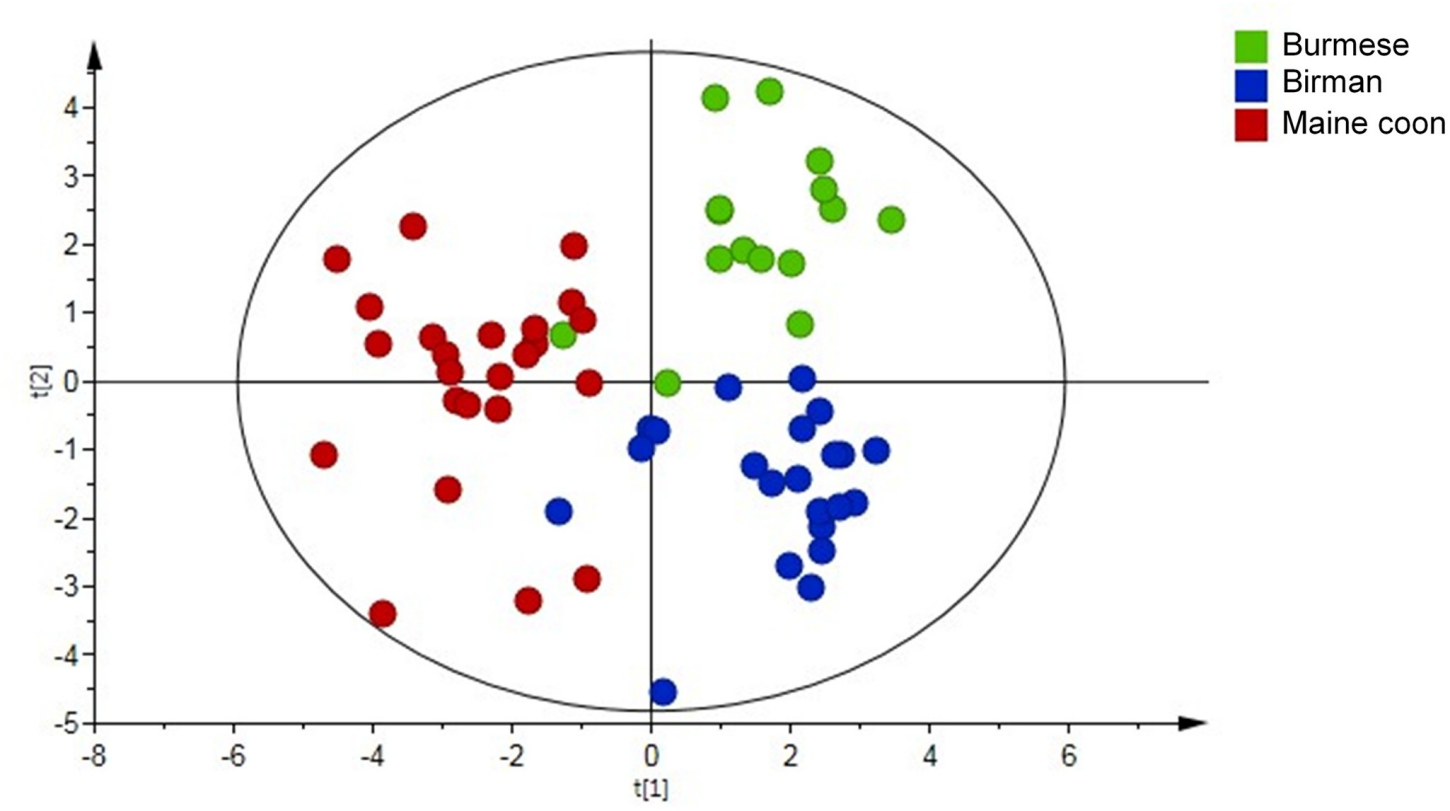
Partial Least Square Discriminant Analysis (PLS-DA) score plot derived from Nuclear Magnetic Resonance (NMR)-based metabolomics analysis in 63 cats by breed; Burmese (n = 15), Birman (n = 23) and Maine coon (n = 25) cats.

A significant PLS-DA model (Fig 1) successfully separated the breeds: the first component separated MCO cats from Burmese and Birman, while the second component separated Burmese from Birman. Out of 58 quantified metabolites 21 were found discriminative based on their VIP along the first and second component. The discriminative metabolites were subjected to univariate statistical analysis followed by correction for multiple testing and 18 metabolites were reconfirmed (Table 3). The Burmese breed was characterized by higher levels of the branched-chain amino acid (BCAA) valine, the aromatic amino acid tyrosine, the amino acid metabolite 2-oxoisocaproic acid, and acetylcarnitine compared to MCO cats. Additionally, 2-hydroxybutyric acid was higher and acetic acid was lower relative to the Birman breed. Lysine and O-phosphocholine levels were lowest in Burmese cats and significantly different from both other breeds. The metabolic fingerprint of MCO cats was characterized by high concentrations of arginine, asparagine, methionine, creatine, dimethylglycine, succinic acid and low levels of acetylcarnitine, carnitine and tyrosine compared to Burmese and Birman cats. Birman cats showed high levels of creatinine and low taurine concentrations compared to Burmese and MCO cats.

**Table 3:** Metabolites found different between the three cat breeds in 63 cats.

	Burmese	MCO	Birman	VIP	VIP	*P*^3^
	(n = 15)	(n = 25)	(n = 23)	comp 1^2^	comp 2^2^	
Metabolite	Concentration (μM)^1^			(95% CI)	(95% CI)	
**2-Oxoisocaproic acid**	5.5±0.9 ^a^	4.3±0.9 ^b^	4.9±1.6 ^a,b^	1.18 (0.39)	1.16 (0.57)	0.008
**Acetic acid**	7.7 ^a^	10.1 ^a,b^	11.3 ^b^	-	1.41 (0.62)	0.002
	(5.0–10.0)	(8.7–12.6)	(9.7–17.0)			
**Acetylcarnitine**	4.1 ^a^	2.6 ^b^	3.6 ^a^	1.88 (0.89)	1.6 (0.47)	<0.001
	(3.0–5.1)	(2.2–3.1)	(2.8–4.2)			
**Arginine**	122.1±19.0 ^a^	146.7±22.4 ^b^	118.2±13.0 ^a^	2.14 (0.53)	1.67 (0.37)	<0.001
**Asparagine**	57.9 ^a^	68.2 ^b^	60.6 ^a^	2 (0.99)	1.55 (0.85)	<0.001
	(50.1–66.8)	(63.3–72.9)	(53.2–62.7)			
**Carnitine**	25.0 ^a^	21.7 ^b^	25.6 ^a^	1.46 (1.04)	1.13 (0.86)	0.004
	(23.9–28.4)	(18.3–28.5)	(24.3–31.1)			
**Creatine**	9.5 ^a^	12.2 ^b^	8.4 ^a^	1.23 (1.09)	-	0.001
	(6.3–13.7)	(10.6–23.4)	(7.4–12.3)			
**Creatinine**	128.8±28.7 ^a^	123.7±19.0 ^a^	146.8±19.0 ^b^	1.5 (0.87)	1.57 (0.97)	0.002
**Dimethylglycine**	4.1 ^a^	5.0 ^b^	4.1 ^a^	1.5 (1.25)	1.26 (0.82)	0.001
	(3.5–4.3)	(4.5–6.4)	(3.7–4.7)			
**Histidine**	110.5±16.3 ^a,b^	120.7±14.3 ^a^	107.2±16.4 ^b^	-	1.03 (1.02)	0.012
**Lysine**	72.0 ^a^	107.7 ^b^	114.3 ^b^	-	1.81 (1.13)	<0.001
	(63.6–80.2)	(93.4–138.9)	(85.2–126.2)			
**Methionine**	40.3±9.0 ^a^	48.1±9.0 ^b^	35.0±7.9 ^a^	1.99 (1)	1.68 (0.66)	<0.001
**O-Phosphocholine**	3.3±0.5 ^a^	4.1±0.9 ^b^	4.2±0.7 ^b^	-	1.69 (1.11)	0.001
**Succinic acid**	15.0 ^a^	17.7 ^b^	14.6 ^a^	1.63 (1.32)	1.27 (1.13)	0.001
	(12.2–17.1)	(16.2–19.9)	(10.2–17.6)			
**Taurine**	295.8±86.4 ^a^	286.6±121.5 ^a^	193.3±89.2 ^b^	1.93 (0.91)	1.3 (0.8)	0.003
**Tyrosine**	60.7±10.7 ^a^	47.2±10.4 ^b^	59.3±10.2 ^a^	1.28 (0.78)	1.51 (0.75)	<0.001
**2-Hydroxybutyric acid**	20.0±4.7 ^a^	17.0±4.2 ^a,b^	16.1±3.6 ^b^	-	1.28 (0.99)	0.020
**Valine**	183.0±28.0 ^a^	156.2±34.2 ^b^	176.5±36.5 ^a,b^	-	1.11 (0.58)	0.032

VIP, variable influences of projection; CI, confidence interval; MCO, Maine coon.

^1^ Concentrations are shown as mean with standard deviation for normally distributed data, and as median and interquartile range for non-normally distributed data.

^2^ VIPs (95% CI) are based on the PLS-DA model presented in Fig 1.

^3^ *P*-values represent breed differences based on univariate statistical analyses (one-way ANOVA or Kruskal-Wallis test), *P* < 0.013 was considered significant based on the Benjamini-Hochberg correction for multiple testing.

^a,b^ Numbers within a row with different superscript letters differ from another at *P* < 0.05, based on univariate statistics including Tukey’s post hoc test.

Each point represents one cat. Model parameters: R2X component 1 = 0.103, R2X component 2 = 0.074, R2Y(cum) = 0.668, Q2(cum) = 0.449; Cross-validated ANOVA: *P* < 0.001. Significant metabolites are presented in Table 3.

Univariate regression analysis including adjustments for BW and BCS for each NMR metabolite identified as discriminative between breeds, revealed that breed remained as a significant explanatory variable for the following 15 metabolites: 2-hydroxybutyric acid, 2-oxoisocaproic acid, acetic acid, acetylcarnitine, arginine, asparagine, creatinine, dimethylglycine, histidine, lysine, methionine, O-phosphocholine, succinic acid, taurine, and tyrosine (Table 4). For the metabolites carnitine, creatine, and valine, breed did not remain significant, and the discriminant effect was instead due to BW, BCS or a combination of the explanatory variables. Arginine and creatine concentrations increased with 4.6 μM (*P* = 0.04) and 31.6% (*P* < 0.0001) per kg BW, respectively. Tyrosine concentrations were affected by BCS, with overweight cats having higher average concentrations than normal weight (*P* = 0.042) and underweight (*P* = 0.013) cats.

**Table 4:** Metabolites remaining discriminant for breeds after adjusting for body weight and body condition score.

Metabolite	Concentration (μM)^1^			*P*^2^
	Burmese	Maine coon	Birman	
	(n = 15)	(n = 25)	(n = 23)	
**2-Hydroxybutyric acid**	20.5 (17.9–23.1) ^a^	17.1 (15.1–19.1) ^b^	16.8 (14.7–19.0) ^b^	0.033
**2-Oxoisocaproic acid**	5.8 (5.0–6.5) ^a^	4.3 (3.7–4.9) ^b^	4.9 (4.3–5.6) ^a,b^	0.014
**Acetic acid**	8.4 (5.6–11.2) ^a^	10.9 (8.7–13.1) ^a,b^	13.4 (11.0–15.8) ^b^	0.011
**Acetylcarnitine**	4.1 (3.5–5.8) ^a^	2.8 (2.5–3.2) ^b^	3.5 (3.1–4.0) ^a^	0.003
**Arginine** *	124.0 (113.0–134.9) ^a^	141.6 (133.0–150.1) ^b^	124.3 (115.0–133.6) ^a^	0.022
**Asparagine**	57.0 (52.3–62.2) ^a^	68.7 (64.1–73.5) ^b^	58.4 (54.3–62.9) ^a^	0.003
**Creatinine**	126.3 (113.6–139.0) ^a^	120.0 (110.1–130.0) ^a^	150.5 (139.7–161.3) ^b^	0.0002
**Dimethylglycine**	3.7 (3.2–4.3) ^a^	4.9 (4.4–5.5) ^b^	4.3 (3.8–4.9) ^a,b^	0.013
**Histidine**	111.8 (102.2–121.5) ^a,b^	123.5 (115.9–131.0) ^a^	107.3 (99.1–115.5) ^b^	0.027
**Lysine**	70.1 (60.6–81.0) ^a^	104.1 (92.9–116.6) ^b^	110.1 (97.3–124.5) ^b^	<0.0001
**Methionine**	39.7 (34.8–45.3) ^a^	47.8 (43.1–53.0) ^b^	34.3 (30.7–38.4) ^a^	0.0005
**O-phosphocholine**	3.1 (2.7–3.6) ^a^	4.1 (3.8–4.5) ^b^	4.1 (3.7–4.5) ^b^	0.0004
**Succinic acid**	13.0 (11.1–15.2) ^a^	18.0 (15.9–20.4) ^b^	13.0 (11.4–14.9) ^a^	0.002
**Taurine**	283.3 (218.7–347.9) ^a^	292.8 (242.2–343.3) ^a^	184.2 (129.3–239.1) ^b^	0.007
**Tyrosine** ^x^	56.5 (50.3–63.4) ^a^	44.8 (41.0–49.1) ^b^	58.5 (53.0–64.5) ^a^	0.001

^1^ Concentrations (μM) are shown as estimated least square means for each breed, with 95% confidence intervals (CI). If residuals were not normally distributed, data were logged for analysis, and least square means were back-transformed and reported as geometric means, with 95% CI.

^2^ *P*-value for type 3 tests of breed as fixed effect.

^a,b^ Numbers within a row with different superscript letters differ from another at *P* < 0.05.

* For arginine there was an additional effect from body weight (*P* = 0.04).

^**x**^ For tyrosine there was an additional effect from body condition (*P* = 0.03).

## Discussion

In the present study the metabolic fingerprint was compared between three breeds (Burmese, MCO and Birman cats) and higher concentrations of biomarkers associated with insulin resistance and/or diabetes were observed in Burmese cats, the breed with highest risk of developing DM. Breed differences were also evident from serum biochemistry and hormone assays, where breed was an important individual variable explaining the variation in the data.

In people, BCAAs (isoleucine, leucine, valine) and the aromatic amino acids tyrosine, and phenylalanine have been identified as early biomarkers for T2DM [39]. In the present study, the initial comparison of the metabolic fingerprint of the three cat breeds also showed higher levels of valine, leucine, tyrosine, 3-methyl-2-oxovaleric acid (a breakdown product of isoleucine) and 2-oxoisocaproic acid (a breakdown product of leucine) in Burmese cats. After adjustment for body weight and body condition score only tyrosine and 2-oxoisocaproic acid remained significantly different from the MCO breed, suggesting that some of the metabolic differences are due to a high proportion of Burmese cats being overweight. Tyrosine has been shown to be associated with T2DM and insulin resistance in people [39, 40, 54] in epidemiological studies. Mechanistic studies suggest that 2-oxoisocaproic acid induces insulin secretion in rat islets [55]. Alternatively, high levels of 2-oxoisocaproic acid, indicative of impaired BCAA metabolism, may lead to an accumulation of toxic intermediates which have been suggested to cause mitochondrial stress and impaired insulin action [56]. The higher concentrations of insulin in Burmese than in MCO cats may indicate insulin resistance and thus be related to the increased risk for DM.

Also dimethylglycine, an amino acid derivative, differed in concentration between breeds, with Burmese cats having lower concentrations than MCO cats, after adjustment for body weight and body condition score. Low plasma levels of dimethylglycine have been associated with higher blood glucose levels in human [57]. Additionally, higher concentrations of 2-hydrobutyric acid were observed in the Burmese compared to the MCO and Birman breed. 2-hydroxybutyric acid, derived from alpha-ketobutyrate which is produced by glutathione anabolism and amino acid catabolism (threonine and methionine), is an early marker for insulin resistance and impaired glucose regulation in people, and the underlying mechanism may involve increased lipid oxidation and oxidative stress [58].

Acetylcarnitine, a short-chain acylcarnitine, was significantly higher in Burmese and Birman cats than in MCO cats. Acetylcarnitine has been shown to be higher in T2DM patients than in healthy controls, and high acylcarnitines have also been associated with IR in people [59]. Acetylcarnitine concentrations were significantly correlated with plasma HbA1c in people, which suggests that higher acetylcarnitine levels are associated with an increasing severity of diabetes [60]. Experimental administration of acetylcarnitine improves insulin-mediated glucose disposal [61]. Acylcarnitines are also involved in an alternative model for explaining the obesity-induced IR focusing on intra-mitochondrial disturbances. According to this theory, an overload of lipids cause an increase, rather than a decrease, in beta-oxidation, leading to production and accumulation of acylcarnitines, which in turn interfere with insulin signaling in skeletal muscle [20, 62]. Indeed, VLDL-TG were significantly higher in Burmese and Birman compared to MCO cats, although the BCS was lower in both Birman and MCO than in Burmese cats.

The concentrations of adiponectin were lowest in Burmese cats, but significantly different only from the MCO breed. Adiponectin is an adipocyte-derived hormone, with an inconsistent association with obesity in cats. Some studies have shown a negative correlation [50, 63–66], while others did not detect associations between total adiponectin concentrations and obesity [49, 67]. Increasing BW in our study was associated with lower adiponectin concentrations, however, we did not identify BCS as an independent factor influencing adiponectin levels. The three breeds in this study represent two normal-sized cat breeds, the Burmese and the Birman, and one large-sized breed, the MCO. Adiponectin directly regulates glucose metabolism and increases insulin sensitivity in people by stimulating fatty-acid oxidation, glucose uptake, and reduces gluconeogenesis in the liver [27, 68]. In cats and people with DM, adiponectin concentrations are even lower than in overweight and obese individuals, indicating that the degree of hypoadiponectinemia is more closely related to the degree of insulin resistance than to the degree of adiposity [25, 26, 69, 70].

The plasma metabolic profile of MCO cats was characterized by higher concentrations of two essential amino acids, arginine and methionine [71], and one non-essential amino acid, asparagine, compared to Burmese and Birman cats. Arginine is essential due to its crucial role in the urea cycle to excrete ammonia. Methionine, a sulfur containing proteinogenic amino acid, is needed as methyl-group donor and acceptor.

Breed differences for plasma biochemical analytes in cats have been reported previously, with Birman cats displaying higher creatinine and total protein concentrations than other cats [72]. Birman cats had significantly higher creatinine concentrations than the Burmese and MCO cats also in the present study. The reasons for these findings are unclear. Creatinine was also associated with BCS, with higher concentrations seen in overweight cats than in both normal weight and underweight cats. Serum creatinine is a byproduct of muscle metabolism, and it is possible that the underweight cats in our study had less muscle mass which might explain part of the effect of BCS on creatinine.

The present study is the first using a metabolomics approach to assess differences between cat breeds, with the comparatively large number of samples being a strength. Metabolomics uses relatively cheap and noninvasive techniques to produce large amounts of data and thus shows potential to improve disease diagnostics [73]. In the search for biomarkers, many metabolites can be measured, and once a biomarker is identified and validated, other simpler methodologies can be used in a clinical setup. An improved understanding of the variation in metabolism between different breeds may thus enable identification of new markers related to abnormal metabolism/insulin resistance and potentially facilitate the development of therapies to improve glucose tolerance in cats of high-risk breeds. Although DM in people shows great heterogeneity not only between but also within different types, and not all variants of T2DM may have feline counterparts [74], the results also support the use of the cat as a model for T2DM in people.

## Conclusions

To our knowledge, this is the first study including NMR data from a comparably large cohort of healthy cats of three breeds with different risk of developing DM; the Burmese, MCO and Birman. We found significant differences in the metabolic profiles between the included cat breeds, based on an NMR metabolomics approach, serum biochemistry analyses and hormone immunoassays. Our results indicate that Burmese cats have a metabolic fingerprint similar to that in people with IR. An improved understanding of the variation in metabolism between different breeds may facilitate the development of therapies to improve glucose tolerance in cats of high risk breeds.
